# The management of appendicular abscesses in a Tunisian Tertiary Care Hospital

**DOI:** 10.1186/s12893-021-01424-8

**Published:** 2021-12-18

**Authors:** Atef Mejri, Khaoula Arfaoui, Mohamed Ali Mseddi, Mohamed Ben Slima, Sarra Saad, Marwen Yahyaoui

**Affiliations:** 1 Department of General Surgery, Jendouba Hospital, Jendouba, Tunisia; 2 Faculty of Medicine of Tunis, Tunis El Manar University, Tunis, Tunisia; 3 Department B of General Surgery, Rabta Hospital, Tunis, Tunisia

## Abstract

**Background:**

This study aims to describe the epidemiological, clinical, and radiological features of appendicular abscesses, compare the different approaches, and assess the safety and utility of laparoscopy in its management.

**Methods:**

This descriptive retrospective study was carried out over 3 years extending from January 2017 to December 2019, reporting 150 appendicular abscesses cases. Data were collected from the register of the general surgery department B of the Rabta hospital. Cases with appendicular abscess were included. Files concerning patients with early appendicitis, non-appendicular abscesses or generalized appendicular peritonitis were excluded. Data were analysed with Statistical Package for the Social Sciences (SPSS) software. In univariate analysis, we used the chi^2^ test, the Fischer test, the Student t test. The factors retained by the univariate analysis were introduced into a logistic regression model. The significance level was set to 0.05.

**Results:**

The mean age was 40.51 years. The gender ratio in patients with appendicular abscesses was M:F 1.94:1. Pain in the right iliac fossa associated with fever was the most common symptom (78% of cases). Clinical examination showed tenderness in the right iliac fossa in 38% of cases, rebound and guarding were found in 77 patients (51.3%), and a palpable mass was noted in 4 cases (4.2%). Imaging was done to confirm diagnosis; 46 patients underwent ultrasonogram and this confirmed the diagnosis in 26 patients (56%), while among the 71 patients who underwent CT abdomen confirmatory diagnosis was made in 65 patients (91.55%). An appendectomy was performed in 148 patients (98.6%) via laparoscopic approach in 94 patients, open Mac burney procedure in 32 cases (21.3%) and midline incision in 24 cases (16%). Two patients had an ileocecal resection. The appendix was most commonly located retrocecally (55.3%) in our cohort. The laparoscopic approach was performed in 94 patients (62.6%), and we had to convert in 44 patients due to dissection difficulties (46.8%). Among patients who underwent laparascopic approach 7 had developed peritonitis.. There were only 2 deaths. The mortality rate was 0,013%. The median duration of outpatient followup was 6 months (4–24 weeks) and was uneventful.

**Conclusion:**

Appendicular abscess is a disease of young adults more common in men. Location of the appendix in our case series was mostly retroceacal. Laparoscopy was associated with good outcomes; peritonitis was uncommon and mortality was rare. The laparoscopic approach is a safe surgical technique for treating appendicular abscess and it can be considered as the routine approach for this condition In developing countries with limited technical resources, laparoscopy guarantees the absence of recurrence, reduces healthcare costs and decreases the risk of treating a severe disease conservatively.

## Introduction

Appendicular abscesses represent 2–7% of complicated acute appendicitis [1]. Although the laparoscopic approach is the gold standard of management in uncomplicated appendicitis, appendicular abscesses' treatment modalities remain controversial [2, 3]. This work aims to study the epidemiological, clinical,and radiological features of appendicular abscesses and assess the place of laparoscopy in its management.

## Methods

Our study is descriptive retrospective analysis performed over three years, from January 2017 to December 2019, in patients hospitalized for an appendicular abscess. We used a data source: the register of the visceral and digestive surgery department B of the Rabta Hospital and patients' clinical records.The search term used were << acute appendicitis >> 0.150 cases of appendicular abscess were collected out of 1190 acute appendicitis. The age cutoff was 14 years old.

We excluded from our study: Patients with early appendicitis, suppurative appendicitis, non-appendicular abscesses in the right iliac fossa, localized peritonitis in the right iliac fossa without abscess, and acute generalized peritonitis.

All patients underwent urgent surgical intervention combined with necessary intensive care measures. Written informed consent obtained from patients and from legally authorized representative of minors.

Ethical approval for the study was obtained from the Committee for Medical Ethics at the Rabta Hospitalunder number CEM S03-01/2021.

Statistical analyses were performed using the SPSS statistical package for Windows version 20.

## Results

**Fig. 1:**
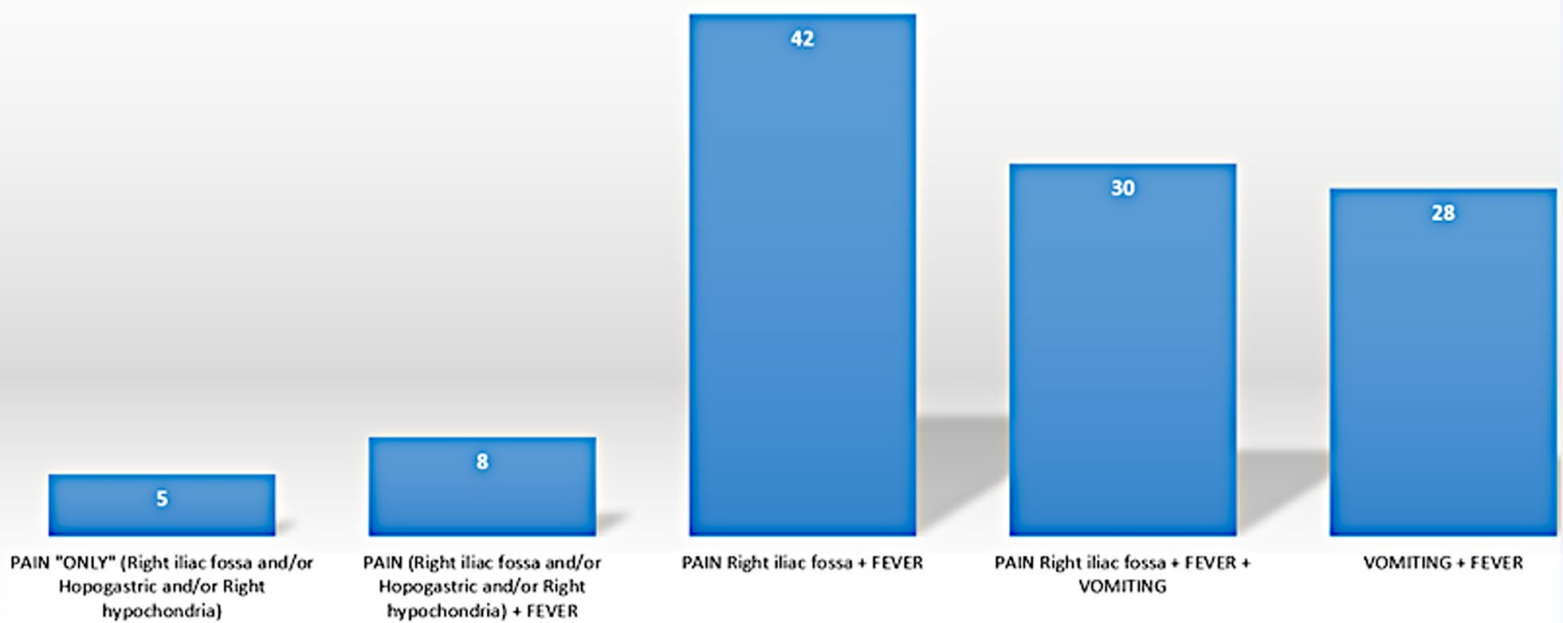
Symptoms incidence among the patients

Among the 1190 cases of acute appendicitis operated in our center during the study, only 150 (12.6%) were complicated with appendicular abscess. The mean age was 40.51 years, ranging from 15 to 89 years. The gender ratio in patients with appendicular abscesses is M:F 1.94:1.The primary symptom was abdominal pain (100% of cases) localized in the right iliac fossa in 95% of cases. Pain in the right iliac fossa associated with fever was the most common symptom association (78% of cases) (Fig. 1).Clinical examination showed tenderness in the right iliac fossa in 38% of cases, rebound and guarding were found in 77 patients (51.3%), and a palpable mass was noted in 4 cases (4.2%). Leukocytosis was noted in 149 cases (99.3%), with an average of 16,438 cells /Cu mm (Table 1). An increase in the C-reactive protein level was noted in 148 patients (98.7%), with an average of 163.7 mg/l. Imaging was done to confirm diagnosis; 46 patients underwent ultrasonogram and this confirmed the diagnosis in 26 patients (56%), while among the 71 patients who underwent CT abdomen confirmatory diagnosis was made in 65 patients (91.55%).

**Table 1:** Laparo-conversion incidence among the patients

Laparoscopic approach, n = 94 patients		
	Laparo-conversion n = 44 (%)	Laparoscopy without conversion n = 50 (%)
Symptoms delay		
< 3 days	12 (31.5)	26 (68.5)
3–7 days	12 (42.8)	16 (57.2)
> 7 days	20 (71.4)	8 (28.6)
Signs		
Guarding right iliac fossa	35 (71.4)	14 (28.5)
Tenderness right iliac fossa	12 (31.5)	26 (68.5)
Mass right iliac fossa	3 (75)	1 (25)
CT scan abscess diameter > 5 cm		
	29 (70.7)	12 (29.3)
Intraoperatively findings		
Retrocecal appendix	37 (71.1)	15 (28.9)
Mesoceliac appendix	3 (100)	0 (0)
Agglomerate small intestine	48 (63.16)	28 (36.84)
False membranes	27 (69.2)	12 (30.8)

The appendicular abscess size on CT scan varied between 1 and 14 cm with an average of 4 cm. An appendectomy was performed in 148 patients (98.6%), and two patients had an ileocecal resection. Intraoperatively the most common location of the appendix was retrocaecal (55.3%).The other locations were preileal(28%), sub coecal (8%), pelvic (5.3%) and postileal (3.3%).Anagglomerate of small intestinal loops with severe adhesions was found in 112 patients (74.7%), false membranes were noted in 67 patients (44.7%), and anappendicular mass was noted in 5 patients.

The laparoscopic approach was performed in 94 patients (62.6%), and we had to convert in 44 patients for dissection difficulties (46.8%). Conversion into open procedure was conducted via Mac burney incision in 17 cases (39%) and medline incision in 27 cases (61%). Residents conducted 32 (72%) laparo-conversion out of 44. Table 1 shows the predictors of laborious dissection that led to laparo-conversion. In the laparoscopic approach's cases, the average operative time was 120 min ranging from 40 to 220 min. The mean hospital stay after the laparoscopic approach was 4 ± 1.3 days, with extremes ranging from 2 to 10 days. Among patients who underwent laparascopic approach 7 had developed localized peritonitis in the right iliac fossa. Six patients required second emergency surgery performed laparoscopically, and one patient had a conservative medical treatment based on intensive antibiotic therapy. All seven patients had a good outcome. There were two deaths for all cases; due to a massive pulmonary embolism after a laparoscopic approach and one case of hypoxemic pneumonitis after open surgery. The mortality rate in our study cohort was 0.013%. The median duration of outpatient follow up was 6 months (4–24 weeks) and it was uneventful.

## Discussion

The appendicular abscess is a complication of diagnostic delay, mainly related to atypical clinical presentation of acute appendicitis or a delay in consulting a doctor exceeding 72 h from the onset of symptoms [1, 4]. The most affected group includes adults aged 30–49 years [4, 5]. Although abdominal pain located in the right iliac fossa is a common complaint, most patients presented with a combination of symptoms (pain, fever, and vomiting). 78% (n = 117) of patients in our series presented with an association of Pain in the right iliac fossa and fever, and 51.3% (n = 77) had a guarding in the right iliac fossa. The abdominal CT scan is the diagnostic modality of choice especially when there is a hypodense collection, with a contrast-enhancement in the wall, within which the perforated appendix maybe challenging to identify [3, 6, 7]. However, Vons et al. report that 18% of CT scans performed for acute appendicitis don't corroborate intraoperative findings [8]. To overcome diagnostic difficulties, Atema et al. report a score (specificity of 94.7%) for diagnosing complicated acute appendicitis, combining the clinical and radiological findings [9].

The optimal management of appendicular abscesses remains controversial. The conservative method of using a broad spectrum antibiotics and intravenous rehydration therapy often results in total destruction or atrophy (with an obliterated lumen) of the appendix. With conservative method there no risk surgery [4, 10, 11]; we should keep in mind that the failure rate with conservative method exceeds 14% and exposes the patient to risk of peritonitis, which carries more morbidity and mortality [12–15]. Moreover, 50% of patients may suffer a recurrence of their appendicitis following discharge from hospital [14, 16]. Other major issue with the conservative management is the 15% risk of misdiagnosing conditions like intussusception and caecal carcinoma these, when treated conservatively, add considerable morbidity [12, 14, 16].

Certainly, early appendectomy overcomes all the disadvantages of conservative management. However, the intraoperative risks should not be underestimated. Performing the laparoscopic approach for an appendicular abscess is not a unanimous choice [17–19]. Indeed, Horwitz et al. and others have suggested avoiding the laparoscopic approach in complicated appendicitis because of the increased risk of intraoperative complications and postoperative intra-abdominal abscesses [12, 20]. In contrast, the most recent studies advocate the laparoscopic approach in appendicular abscess in view of all the well-known advantages of laparoscopy including a lower incidence of post-operative wound infection and respiratory complications [21] of conversion to open surgery. In fact, it was higher in our series among residents undergoing training than among senior surgeons. Out of the 44 laparo-conversion cases, 32 (72%) were conducted by residents.These findings agree with the data in the literature. So et al. noted a conversion rate of 63% for inexperienced surgeons against 8% for experienced surgeons who performed more than 20 similar surgeries [18, 22, 23]. The reports of Horvath et al. and Katsuno et al. where all appendicular abscesses were operated by laparoscopy, have proven that there is an inverse relationship between the surgeon's experience and specific complications when the laparoscopic approach is performed [18, 22].

In our series, the radiological abscess of more than 5 cm diameter in CT scan and the delay of consultation more than 7 days predicted a dissection difficulty and laparoconversion in respectively 70.7% and 71.4%. Furthermore, the appendicular mass and the retro-caecal appendix(intraoperatively) led to dissection difficulty and conversion in respectively 75% and 71.1%. Our results corroborate with the literature [23–25].

## Conclusion

Despite the fact that complicated appendicitis should be suspected irrespective of age, the current study confirms that this potentially lethal complication shows higher prevalence among males and young adults. Right lower-quadrant pain remains the most common clinical feature associated with fever in our cohort. A palpable mass is not always present and the clinical presentation may not have any distinctive patterns from uncomplicated acute appendicitis. The retrocoecal position of the vermiform appendix is exceedingly common in appendicular abscess and this could be related to the enigmatic presentation and the insidious course of retrocaecal acute appendicitis leading to misdiagnosis and abscess formation. Abdominal CT scan is of great value to confirm the diagnosis and an abscess diameter exceeding 5 cm is highly associated with dissection difficulties. Laparoscopic approach is a safe procedure with a satisfactory success rate and should then be preferred especially in presence of surgeons with high surgical technical skills.

## Data Availability

There are no additional data available to share with the readers. The datasets used and/or analyzed during the current study are available from the corresponding author on reasonable request.

## Abbreviations

SPSS: Statistical Package for the Social Sciences
CT: Computed tomography

## References

[1] Reddy LM, Bai VR, Lakshmi VV (2020). A study on need of emergency laparascopic appendicectomy for appendiceal masses. IJCMSR..

[2] Ismail I, Iusco D, Jannaci M, Navarra GG, Grassi A, Bonomi S (2009). Prompt recognition of stump appendicitis is important to avoid serious complications: a case report. Cases J..

[3] Debnath J, Kumar R, Mathur A, Sharma P, Kumar N, Shridhar N (2015). On the role of ultrasonography and ct scan in the diagnosis of acute appendicitis. Indian J Surg.

[4] Demetrashvili Z, Kenchadze G, Pipia I, Khutsishvili K, Loladze D, Ekaladze E (2019). Comparison of treatment methods of appendiceal mass and abscess: a prospective cohort study. Ann Med Surg (Lond).

[5] Kaya B, Sana B, Eris C, Kutaniş R (2012). Immediate appendectomy for appendiceal mass. Ulusal travma ve acil cerrahi dergisi Turkish J Trauma Emerg Surg..

[6] Lietzén E, Mällinen J, Grönroos JM, Rautio T, Paajanen H, Nordström P (2016). Is preoperative distinction between complicated and uncomplicated acute appendicitis feasible without imaging?. Surgery.

[7] Pinto Leite N, Pereira JM, Cunha R, Pinto P, Sirlin C (2005). CT evaluation of appendicitis and its complications: imaging techniques and key diagnostic findings. AJR Am J Roentgenol.

[8] Vons C, Barry C, Maitre S, Pautrat K, Leconte M, Costaglioli B (2011). Amoxicillin plus clavulanic acid versus appendicectomy for treatment of acute uncomplicated appendicitis: an open-label, non-inferiority, randomised controlled trial. Lancet.

[9] Atema JJ, van Rossem CC, Leeuwenburgh MM, Stoker J, Boermeester MA (2015). Scoring system to distinguish uncomplicated from complicated acute appendicitis. Br J Surg.

[10] Simillis C, Symeonides P, Shorthouse AJ, Tekkis PP. A meta-analysis comparing conservative treatment versus acute appendectomy for complicated appendicitis (abscess or phlegmon). Surgery. 2010; 147(6): 818–29. 10.1016/j.surg.2009.11.013 20149402

[11] Okune EB, Marek G, Jarosaw K. Management of appendiceal mass in children and adults: our experience. Internet J Surg. 2006 (cited 2021 Jan 24);9(2).

[12] Shindholimath VV, Thinakaran K, Rao TN, Veerappa YV (2011). Laparoscopic management of appendicular mass. J Minimal Access Surg.

[13] Surana R, Puri P (1995). Appendiceal mass in children. Pediatr Surg Int.

[14] Bahram MA (2011). Evaluation of early surgical management of complicated appendicitis by appendicular mass. Int J Surg.

[15] Samuel M, Hosie G, Holmes K (2002). Prospective evaluation of nonsurgical versus surgical management of appendiceal mass. J Pediatr Surg.

[16] Senapathi PSP, Bhattacharya D, Ammori BJ (2002). Early laparoscopic appendectomy for appendicular mass. Surg Endosc.

[17] Elkbuli A, Diaz B, Polcz V, Hai S, McKenney M, Boneva D (2018). Operative versus non-operative therapy for acute phlegmon of the appendix: is it safer? A case report and review of the literature. Int J Surg Case Rep.

[18] Horvath P, Lange J, Bachmann R, Struller F, Königsrainer A, Zdichavsky M (2017). Comparison of clinical outcome of laparoscopic versus open appendectomy for complicated appendicitis. Surg Endosc.

[19] Helling TS, Soltys DF, Seals S (2017). Operative versus non-operative management in the care of patients with complicated appendicitis. Am J Surg.

[20] Tartaglia D, Fatucchi LM, Mazzoni A, Miccoli M, Piccini L, Pucciarelli M (2020). Risk factors for intra-abdominal abscess following laparoscopic appendectomy for acute appendicitis: a retrospective cohort study on 2076 patients. Updates Surg.

[21] Quah GS, Eslick GD, Cox MR (2019). Laparoscopic appendicectomy is superior to open surgery for complicated appendicitis. Surg Endosc.

[22] Katsuno G, Nagakari K, Yoshikawa S, Sugiyama K, Fukunaga M (2009). Laparoscopic appendectomy for complicated appendicitis: a comparison with open appendectomy. World J Surg.

[23] So JBY, Chiong E-C, Chiong E, Cheah W-K, Lomanto D, Goh P (2002). Laparoscopic appendectomy for perforated appendicitis. World J Surg.

[24] Wagner PL, Eachempati SR, Aronova A, Hydo LJ, Pieracci FM, Bartholdi M (2011). Contemporary predictors of conversion from laparoscopic to open appendectomy. Surg Infect (Larchmt).

[25] Antonacci N, Ricci C, Taffurelli G, Monari F, Del Governatore M, Caira A (2015). Laparoscopic appendectomy: which factors are predictors of conversion? A high-volume prospective cohort study. Int J Surg.

